# A Review of Genomic Models for the Analysis of Livestock Crossbred Data

**DOI:** 10.3389/fgene.2020.00568

**Published:** 2020-06-26

**Authors:** Joana Stock, Jörn Bennewitz, Dirk Hinrichs, Robin Wellmann

**Affiliations:** ^1^Department of Animal Breeding and Genetics, Institute of Animal Science, University of Hohenheim, Stuttgart, Germany; ^2^Department of Animal Breeding, University of Kassel, Witzenhausen, Germany

**Keywords:** genomic selection, genomic models, livestock, crossbreeding, heterosis

## Abstract

Livestock breeding has shifted during the past decade toward genomic selection. For the estimation of breeding values in purebred breeding schemes, genomic best linear unbiased prediction has become the method of choice. Systematic crossbreeding with the aim to utilize heterosis and breed complementary effects is widely used in livestock breeding, especially in pig and poultry breeding. The goal is to improve the performance of the crossbred animals. Due to genotype-by-environment interactions, imperfect linkage disequilibrium, and the existence of dominance and imprinting, purebred and crossbred performances are not perfectly correlated. Hence, more complex genomic models are required for crossbred populations. This study reviews and compares such models. Compared to purebred genomic models, the reviewed models were of much higher complexity due to the inclusion of dominance effects, breed-specific effects, imprinting effects, and the joint evaluation of purebred and crossbred performance data. With the model assessment work conducted until now, it is not possible to come to a clear recommendation as to which existing method is most suitable for a specific breeding program and a specific genetic trait architecture. Since it is expected that a superior method includes all the different genetic effects in a single model, a dominance model with imprinting and breed-specific SNP effects is proposed. Further progress could be made by assuming realistic covariance structures between the genetic effects of the different breeding lines, and by using larger marker panels and mixture distributions for the SNP effects.

## Introduction

The crossing of different lines or breeds is widely used in animal breeding with the main aim to produce superior offspring. This superiority results from heterosis and from breed complementary effects. Continuous and discontinuous crossbreeding schemes have been designed and are implemented in various livestock species (Lopez-Villalobos et al., 2000; Samorè and Fontanesi, 2016). In discontinuous schemes, crossbred animals are used solely for production and are not selected as parents of the next generation. Breeding takes place in the parental breeds and the breeding goal is usually to improve crossbred performance. The level of organization in such a system is high and it is sometimes difficult to utilize by-products, such as male offspring of mother lines. These schemes can be predominantly found in livestock species with a high female reproduction rate such as pigs and poultry. In continuous breeding schemes, the female crossbreds are used as parents to breed the next generation. These systems are sometimes implemented in livestock species with a low female reproduction rate such as cattle. Since there are substantial non-additive effects for reproduction traits in dairy cattle (Jiang et al., 2017), the aims of crossbreeding in dairy cattle are to improve reproduction traits and other functional traits by exploiting heterosis and imprinting and by removing inbreeding depression (Sørensen et al., 2008; Buckley et al., 2014).

A further form of crossbreeding is the upgrading of low-performance breeds with high-yielding breeds. This introgression of genes from high-yielding breeds increases the production level in subsequent generations and reduces inbreeding depression by increasing the genetic diversity of the low-performance breed. This breeding system has frequently been applied to local breeds, such as the German Vorderwald cattle (Hartwig et al., 2014, 2015). However, if upgrading is repeated over several generations, then the breed eventually goes extinct because the native alleles are removed from its gene pool. The formation of a synthetic breed can also be seen as a special form of crossbreeding. A well-known example is the establishment of the so-called Schwarzbuntes Milchrind in the former East Germany (Freyer et al., 2008).

Livestock breeding has shifted toward genomic selection, which is now frequently implemented in large pure breeds. The core of the system that has been implemented in pure breeds is a reference population that consists of genotyped and phenotyped animals. The phenotypes are either the animal's own performance records, or deregressed conventional breeding values. The reference population is needed for the prediction of marker effects. The marker effects are then used for predicting genomic breeding values of the genotyped selection candidates. The reliability of genomic breeding values depends on the size of the reference population, on the effective number of chromosome segments, and on the method used for the prediction of marker effects (Goddard, 2009).

Extensive research has been dedicated to develop statistical models for the prediction of marker effects. These statistical models include the SNP-BLUP model that assumes normally distributed SNP effects, various Bayesian models that assume more heavy-tailed distributions, as well as non-parametric and semi-parametric models (Meuwissen et al., 2001; Gianola, 2013). More complex models assume different SNP variances, depending on the type of control region the SNP belongs to MacLeod et al. (2016). Some models avoid the prediction of marker effects by building a genomic relationship matrix based on SNP genotypes. The most prominent method based on genomic relationships is GBLUP, which is an equivalent model to SNP-BLUP (VanRaden, 2008; Goddard, 2009). The genotyped selection candidates are included in the model, and their genomic breeding values are calculated by utilizing their genomic relationships with the reference population. GBLUP assumes that all animals are genotyped, which is in general not the case. Therefore, the genomic breeding values are blended in a second step with pedigree-based breeding values to obtain genomically enhanced breeding values on which selection decisions are based. This two-step procedure can be avoided with so-called single-step GBLUP models (ssGBLUP). They were developed as extensions of GBLUP. Single-step models include genotyped and non-genotyped animals simultaneously (Legarra et al., 2009, 2014; Aguilar et al., 2010; Christensen and Lund, 2010) and assume a particular covariance structure for the breeding values that is computed from genomic and pedigree-based relationships. Fernando et al. (2014) extended the single step model toward non-normally distributed marker effects. In purebred routine application mostly additive effects are considered, with dominance being an integral part of the estimated breeding values. Some genomic models were extended toward accounting for dominance explicitly, but this increased the realibilities of the breeding values only slightly (Su et al., 2012; Wellmann and Bennewitz, 2012; Azevedo et al., 2015).

To summarize, it seems that in practical purebred genomic evaluations, GBLUP and ssGBLUP have and will become the models of choice, and non-additive gene effects are usually not an issue. The picture is however somewhat different if data from crossbred animals in combination with the parental purebred data is analyzed. The potential applications of genomic models with non-additive genetic effects have been reviewed by Varona et al. (2018). The main breeding goal is in this case to improve the performance of the crossbred animals. Due to genotype-by-environment interaction, imperfect LD, and the existence of dominance, epistasis and imprinting, purebred and crossbred performances (PP and CP, respectively) are not perfectly correlated (e.g., Wei and van der Werf, 1995; Dekkers, 2007; Zumbach et al., 2007; Duenk et al., 2019). Wientjes and Calus (2017) reviewed existing literature about purebred-crossbred correlations in pigs. The average from 201 reported correlation coefficients was 0.63 with 50% of the reported coefficients being between 0.45 and 0.87. The purebred-crossbred correlation affects the optimal design of the reference population (van Grevenhof and van der Werf, 2015) and the choice of an appropriate genomic model.

While genomic models are well-established for pure breeds, much research has been conducted in the recent years to develop genomic models for the analysis of crossbred data. The aim of this study is to review genomic models for the prediction of crossbred performance that were recently developed and were evaluated either using simulated or real crossbred data.

## Genomic Models

Genomic models for crossbred data are extensions of purebred models. The extensions were made in several directions. Most genomic models for the analysis of crossbred data are developed for two-way crosses. A two-way cross X is created from a sire line A and a dam line B, which are usually not inbred. The pure lines have breeding values $a_{A}$ and $a_{B}$ for PP, and breeding values $c_{A}$ and $c_{B}$ for CP. Typically, some animals are genotyped, whereas others are not. The goal is to obtain accurate predictions of the breeding values for CP by utilizing phenotypic information from genotyped and ungenotyped purebred and crossbred animals. An overview over the considered models is given in Table 1.

**Table 1:** Additive and dominance models for the prediction of crossbred performance.

		**Data requirements**		
		**Phenotyped crossbreds**	**Genotyped crossbreds**	
Additive Models	Parental Model	x		
	BSAM/ASGM	x	x	
	Single step	x	(x)	
Dominance Models	Line-independent	(x)	(x)	Provide more accurate breeding values for CP than additive models
	Line-dependent	(x)	(x)	
	Dominance + Imprinting	x	x	

*(x) not necessarily needed but can be utilized*.

The SNP alleles are usually assumed to be biallelic, so they may be coded as alleles 1 and 2. Most authors use centered allele content matrices as proposed by VanRaden (2008). The centering does not affect the predictions, but affects the model-based reliabilities (Strandén and Christensen, 2011). We denote with

$$\begin{array}{l} Z_{G}^{A}=G^{A}-2P^{A} \end{array}$$

the centered allele content matrix for the genotyped animals from line A, whereby the allele content $G_{im}^{A}\in \left\{0,1,2\right\}$ of animal *i* from line *A* is the number of copies of allele 2, animal *i* has at SNP *m*, and $P_{im}^{A}$ is the frequency of allele 2 of SNP *m* in line A. Moreover, we denote with

$$\begin{array}{l} Z_{X}^{A}=G_{X}^{A}-P^{A} \end{array}$$

the centered allele origin matrix for alleles from cross X that originate from line A. That is, $G_{Xim}^{A}\in \left\{0,1\right\}$ is the number of copies of allele 2, crossbred animal *i* has obtained from sire line A at SNP *m*. These matrices are needed to define genetic values of purebred and crossbred animals. The vector with breeding values for CP for animals from line A has the representation

(1) $$\begin{array}{r} \begin{array}{c} c_{A}=Z_{G}^{A}\alpha^{A}, \end{array} \end{array}$$

where $\alpha^{A}$ is the vector with allele substitution effects for CP. The vector with breeding values for PP has the representation

(2) $$\begin{array}{r} \begin{array}{c} a_{A}=Z_{G}^{A}{\tilde{\alpha }}^{A},\; \end{array} \end{array}$$

where ${\tilde{\alpha }}^{A}$ is the vector with allele substitution effects for PP. The equations for $a_{B}$ and $c_{B}$ are similarly.

Most genomic models for two-way crosses utilize, that the vector $a_{X}$ with additive genetic values of the crossbred animals can be decomposed into a contribution $c_{X}^{A}$ that comes from sire line A, and a contribution $c_{X}^{B}$ that comes from dam line B. That is,

(3) $$\begin{array}{r} \begin{array}{c} a_{X}=c_{X}^{A}+c_{X}^{B}, \end{array} \end{array}$$

where

(4) $$\begin{array}{r} \begin{array}{c} c_{X}^{A}=Z_{X}^{A}\alpha^{A},\text{ and }c_{X}^{B}=Z_{X}^{B}\alpha^{B}.\\ \; \end{array} \end{array}$$

The contribution $c_{X}^{A}$ from line A can be further decomposed into a contribution that comes from the breeding values $c_{A}$ for CP, and into a vector $m_{X}^{A}$ that contains the Mendelian sampling terms of the transmitted gametes (Wei and van der Werf, 1994). That is,

(5) $$\begin{array}{r} \begin{array}{c} c_{X}^{A}=0.5\;Z_{\mathrm{XA}}c_{A}+m_{X}^{A}, \end{array} \end{array}$$

where matrix $Z_{\mathrm{XA}}$ assigns animals from line A to their crossbred offspring.

Different models have been developed for predicting CP, which can broadly be classified into additive models and dominance models. While some models predict the breeding values for CP directly with Equation (5), others predict the vector $\alpha^{A}$ with allele substitution effects for CP. In the latter case, the estimated breeding values ${ĉ}_{A}$ for CP in line A are obtained by substituting $\alpha_{\;}^{A}$ with the prediction ${\hat{\alpha }}_{\;}^{A}$ in Equation (1).

### Additive Models

Different additive models have been proposed in the literature. Some models assume that the crossbred animals are genotyped, whereas others do not. The general additive model for a two-way cross is a trivariate model that has two equations for the parental lines, and one equation for the cross. It has the general representation

$$\begin{array}{l} y_{A}=X_{A}b_{A}+Z_{A}u_{A}+a_{A}+E_{A}\\ y_{B}=X_{B}b_{B}+Z_{B}u_{B}+a_{B}+E_{B}\\ y_{X}=X_{X}b_{X}+Z_{X}u_{X}+\ldots +E_{X}, \end{array}$$

where $y_{A},y_{B},y_{X}$ are vectors with phenotypic records of the respective subpopulation, $b_{A},b_{B},b_{X}$ are vectors of fixed effects with design matrices $X_{A},X_{B},X_{X}$, and $u_{A},u_{B},u_{X}$ are vectors of non-genetic random effects with design matrices $Z_{A},Z_{B},Z_{X}$. Finally, $a_{A},a_{B}$ are the breeding values for PP, and $E_{A},E_{B},E_{X}$ are the residual terms. The term “…” in the third equation depends on the respective model.

The first two model equations are needed because PP and CP are genetically correlated (Wientjes and Calus, 2017), so phenotypic records of purebred animals increase the reliabilities of the breeding values for CP.

#### The Parental Additive Model

The parental additive model is based on Equations (2), (3), and (5), and is suitable when the crossbred animals are not genotyped. The model assumes that the Mendelian sampling terms are part of the residuals, so the model equations become

$$\begin{array}{l} y_{A}=X_{A}b_{A}+Z_{A}u_{A}+a_{A}+E_{A}\\ y_{B}=X_{B}b_{B}+Z_{B}u_{B}+a_{B}+E_{B}\\ y_{X}=X_{X}b_{X}+Z_{X}u_{X}+0.5\;Z_{\mathrm{XA}}c_{A}+0.5\;Z_{\mathrm{XB}}c_{B}+E_{X}, \end{array}$$

where $a_{A}=Z_{G}^{A}{\tilde{\alpha }}^{A}$, $a_{B}=Z_{G}^{B}{\tilde{\alpha }}^{B}$, $c_{A}=Z_{G}^{A}\alpha^{A}$, and $c_{B}=\;Z_{G}^{A}\alpha^{A}$.

#### The BSAM and ASGM Model

The model with breed-specific allele effects (BSAM) and the model with breed-independent allele effects, which is also called the across-breed SNP genotype model (ASGM) are based on Equations (2–4), and require that the crossbred animals are genotyped. While the ASGM model predicts one effect per SNP, the BSAM model predicts one effect for the paternal allele, and one for the maternal allele of the crossbred animals. Origin-specific allele effects may occur e.g., due to a different LD pattern between the marker and the QTL, different gene frequencies at the QTL, imprinting effects, or the epistatic effects may be different in the pure breeds. This results in different effects of the marker alleles and thus affects the estimated breeding values.

The first two equations of the BSAM and ASGM model are as above, whereas the third model equation becomes for the BSAM

$$\begin{array}{l} y_{X}=X_{X}b_{X}+Z_{X}u_{X}+Z_{X}^{A}\alpha^{A}+Z_{X}^{B}\alpha^{B}+E_{X}. \end{array}$$

An equivalent representation for the ASGM model is

$$\begin{array}{l} y_{X}=X_{X}b_{X}+Z_{X}u_{X}+a_{X}+E_{X}. \end{array}$$

Ibánez-Escriche et al. (2009) predicted CP of the parental lines from genotyped crossbred animals with BSAM and ASGM, whereby the breed-specific allele substitution effects of the BSAM model were a priori independent. The allele substitution effects were estimated with BayesB, which is a method that assumes that many of them are actually zero. An oligogene trait was simulated with breed-independent QTL effects. Although the SNP effects are expected to be breed-specific due to differences in LD between markers and QTL, the authors found that the BSAM model outperformed ASGM only if the number of markers was low, the number of records for training was high, and if the parental breeds were distantly related.

Lopes et al. (2017) used the BSAM model with normally distributed SNP effects to predict breeding values for CP from crossbred data, and compared the results with conventional GBLUP. The model provided similar prediction accuracies as conventional GBLUP for the traits litter size and gestation length in pigs. It may be not superior to GBLUP because the allele substitution effects of the different breeds were implicitly assumed to be uncorrelated, which is an assumption that is not likely to be fulfilled.

Sevillano et al. (2019) extended the BSAM and ASGM model toward a three-way cross and distinguished SNP that showed a strong trait association from all remaining SNP. For the trait associated SNP breed-specific effects were estimated, whereas for the remaining SNP one effect was estimated, regardless of the allele origin. This model was compared with the BSAM model and with the ASGM model for the trait daily gain by assuming normally distributed SNP effects. Purebred as well as crossbred data was used for training. The results showed a superiority of their method only if the estimated genetic correlations between PP and CP for the trait associated SNPs and the remaining SNPs were unequal.

Vandenplas et al. (2017) derived equations for predicting the reliability of genomic breeding values for CP for BSAM and ASGM models and assumed normally distributed SNP effects. The authors found that BSAM outperformed ASGM for a specific parental line, if the effective number of chromosome segments in the crossbred reference animals that originate from the parental line is less than half the effective number of all chromosome segments that are independently segregating.

#### Additive Single Step Model

While BSAM has the disadvantage that all crossbred animals have to be genotyped, the parental additive model has the disadvantage, that the information provided by the Mendelian sampling terms cannot be utilized for prediction. These problems could be resolved by using a trivariate model of the form

$$\begin{array}{l} y_{A}=X_{A}b_{A}+Z_{A}u_{A}+a_{A}+E_{A}\\ y_{B}=X_{B}b_{B}+Z_{B}u_{B}+a_{B}+E_{B}\\ y_{X}=X_{X}b_{X}+Z_{X}u_{X}+c_{X}^{A}+c_{X}^{B}+E_{X} \end{array}$$

that includes both, genotyped and phenotyped animals. Christensen et al. (2014) derived the joint covariance matrix $A_{A}$ of $c_{X}^{A}$, $c_{A}$, and $a_{A}$ by using the pedigree-based model of Wei and van der Werf (1994) as a starting point. The authors derived the covariance matrix $A_{A}$ from pedigree relationships, and replaced it in a subsequent step by a covariance matrix $H_{A}$ that combines pedigree and genotype information.

Xiang et al. (2016a) validated the model of Christensen et al. (2014) in a two-way pig cross for the trait number of piglets born. The authors found that the inclusion of crossbred genomic information improved the model-based reliabilities for CP and reduced to some extent the bias of prediction.

Tusell et al. (2016) used a single-step model for two-way crossbred pigs and the sire line A, so the model reduced to a bivariate model. The purebred animals were partly genotyped. Since the crossbred animals were not genotyped, the third equation in the model of Christensen et al. (2014) was replaced by a parental additive model equation, i.e., the Mendelian sampling terms were part of the residual. This resulted in a model equation of the form

$$\begin{array}{l} y_{A}=X_{A}b_{A}+Z_{A}u_{A}+a_{A}+E_{A}\\ y_{X}=X_{X}b_{X}+Z_{X}u_{X}+0.5\;Z_{\mathrm{XA}}c_{A}+0.5\;Z_{\mathrm{XB}}c_{B}+E_{X}. \end{array}$$

The authors evaluated six growth and meat traits and found that the genetic correlations between purebred and CP were larger than 0.69 for all traits. The accuracies of the genomic breeding values were higher than those obtained from univariate single-step models that took either purebred or CP into account, and also higher than those obtained with pedigree-based models.

### Dominance Models

Crossbreeding utilizes heterosis and breed complementarity. A widely accepted hypothesis is that heterosis arises predominantly from dominance effects. An animal carries a dominance effect only if it is heterozygous at a particular QTL. We denote with

$$\begin{array}{l} Z_{H}^{X}=H^{X}-{\bar{H}}^{X} \end{array}$$

the centered indicator matrix for heterozygosity. That is, $H_{im}^{X}\in \left\{0,1\right\}$ equals one, if animal *i* is heterozygous at SNP *m*, and ${\bar{H}}_{im}^{X}$ is the heterozygosity of SNP *m* in line X. The dominance model assumes that the vector $g_{X}$ with genotypic values of the crossbred animals has the representation

(6) $$\begin{array}{r} g_{X}=\mu_{X}1+Z_{G}^{X}a^{X}+Z_{H}^{X}d^{X}, \end{array}$$

where $\mu_{X}$ is the population mean, $a^{X}$ is the vector with population-dependent additive effects, and $d^{X}$ is vector with population-dependent dominance effects. The genotypic values of purebred animals are defined accordingly. The trivariate dominance model for a two-way cross and the parental lines has therefore the representation

(7) $$\begin{array}{l} y_{A}=X_{A}b_{A}+Z_{A}u_{A}+Z_{G}^{A}a^{A}+Z_{H}^{A}d^{A}+E_{A}\\ y_{B}=X_{B}b_{B}+Z_{B}u_{B}+\;Z_{G}^{B}a^{B}+Z_{H}^{B}d^{B}+E_{B}\\ y_{X}=X_{X}b_{X}+Z_{X}u_{X}+Z_{G}^{X}a^{X}+Z_{H}^{X}d^{X}+E_{X}, \end{array}$$

which we call the dominance model with line-dependent effects. The vector $c_{A}$ with breeding values for CP from breed A has the representation given in Equation (1), but the vector with allele substitution effects for CP is

$$\begin{array}{l} \alpha^{A}=a^{X}+\left(1-2p^{B}\right)\circ d^{X},\; \end{array}$$

where $p^{B}$ is vector with allele frequencies in the opposite line, and the Hadamard product “∘″ is the component-wise product. The breeding values and allele substitution effects for line B are defined accordingly. Predictions ${â}^{X}$ and ${\hat{d}}^{X}$ of $a^{X}$ and $d^{X}$ are needed to get predictions of the allele substitution effects for CP in line A with equation

$$\begin{array}{l} {\hat{\alpha }}^{A}={â}^{X}+\left(1-2p^{B}\right)\circ {\hat{d}}^{X}. \end{array}$$

Some solvers are unable to account for the fact that $E\left(d^{X}\right)=\mu_{d}^{X}1\neq 0$ for most traits. As shown by Xiang et al. (2016b), one may write $d^{X}=d_{*}^{X}+\mu_{d}^{X}1$ such that $E\left(d_{*}^{X}\right)=0$. Then, the term $Z_{H}^{X}d^{X}$ in Equation (7) equals $Z_{H}^{X}1\mu_{d}^{X}+Z_{H}^{X}d_{*}^{X},$ where $\mu_{d}^{X}$ is treated as an additional fixed effect. The same needs to be done for the parental lines. We can write $Z_{H}^{X}1\mu_{d}^{X}=\mu_{d}^{X}M\left({\hat{h}}_{X}-{\bar{h}}_{X}1\right)$, where *M* is the number of SNPs, ${\hat{h}}_{X}$ is the vector with heterozygosities of the crossbred animals, and ${\bar{h}}_{X}$ is the average heterozygosity of the crossbred animals. Hence, the value $-\mu_{d}^{X}M$ quantifies the inbreeding depression per unit of genomic inbreeding.

Vitezica et al. (2016) demonstrated how dominance models with normally distributed SNP effects can be transformed into equivalent dominance models with animal effects, whereby different covariance matrices are needed for the additive component and the dominance component of the animal effects. That is, if all SNP effects are normally distributed, then the SNP effects model can be replaced by the equivalent animal effects model

$$\begin{array}{l} y_{A}=X_{A}b_{A}+Z_{A}u_{A}+Z_{H}^{A}1\mu_{d}^{A}+\;{\tilde{a}}_{A}+{\tilde{d}}_{A}^{*}+E_{A}\\ y_{B}=X_{B}b_{B}+Z_{B}u_{B}+Z_{H}^{B}1\mu_{d}^{B}+\;{\tilde{a}}_{B}+{\tilde{d}}_{B}^{*}+E_{B}\\ y_{X}=X_{X}b_{X}+Z_{X}u_{X}+Z_{H}^{X}1\mu_{d}^{X}+\;{\tilde{a}}_{X}+{\tilde{d}}_{X}^{*}+E_{X} \end{array}$$

from which the SNP effects can be backsolved. Thereby, the animal effects satisfy ${\tilde{a}}_{X}=Z_{G}^{X}a^{X}$, and ${\tilde{d}}_{X}^{*}=Z_{H}^{X}d_{*}^{X}$, and so on. The joint covariance matrices of the animal effects are given in Christensen et al. (2019).

The SNP effects in Equation (7) were assumed to be line-dependent, which may be the case because the LD between SNP and QTL differs between lines. This may be neglected if the marker panel is sufficiently large. In this case, the SNP effects can assumed to be line-independent, and we obtain the simplified model

$$\begin{array}{l} y_{A}=X_{A}b_{A}+Z_{A}u_{A}+Z_{G}^{A}a+Z_{H}^{A}d+E_{A}\\ y_{B}=X_{B}b_{B}+Z_{B}u_{B}+\;Z_{G}^{B}a+Z_{H}^{B}d+E_{B}\\ y_{X}=X_{X}b_{X}+Z_{X}u_{X}+Z_{G}^{X}a+Z_{H}^{X}d+E_{X}, \end{array}$$

which we call the dominance model with line-independent effects.

Vitezica et al. (2013) emphasized that two different parameterizations of the dominance model exist. The first parameterization, which is given by Equation (6), is suitable for two-way crosses, and includes the additive and dominant SNP effects. In contrast, the second parameterization includes the allele substitution effects and the dominance deviations of the SNP. Both parameterizations are equivalent, but their interpretation is different.

#### Model Evaluation

Zeng et al. (2013) compared a Bayesian dominance model with the corresponding BSAM model and the corresponding ASGM model. A BayesCπ type method was used to estimate the marker effects, so the prior assumption was that the SNP effects are either zero, or come from a normal distribution. The comparison was done for a simulated two-way crossbreeding program. A number of 20 generations of selection was simulated with the aim to improve CP in both parental lines. The marker effects were estimated only once in generation one from crossbred animals and used in all subsequent generations. The simulated traits showed a different degree of dominance variance, ranging from “large” to “realistic,” or null. The dominance model was superior to the BSAM model and to the ASGM model. This superiority depended on the fraction of dominance and thus heterosis in the data, but even for situations where no dominance was simulated, the accuracy of the dominance model was similar to the additive model, indicating the robustness of the model. It can tentatively be concluded, that the use of a dominance model is in general advisable, even if dominance is not an important source of trait variability.

Xiang et al. (2016b) used a dominance model with line-dependent effects for a two-way cross and the parental breeds. The SNP effects were normally distributed, and the additive and dominance effects of the three different populations were correlated. The authors found that the increased predictive ability of the dominance model arose solely from capturing inbreeding depression. This suggests that dominance effects of individual QTL have not been captured. The reason may be that a 60K SNP panel is not sufficient for achieving high LD between markers and QTL, and that the normality assumption is unlikely to be fulfilled.

Esfandyari et al. (2016) compared a Bayesian dominance model with the corresponding Bayesian ASGM model at the example of litter size in a two-way pig cross, whereby BayesC of Habier et al. (2011) was used for prediction. Training was on the parental lines. The prediction accuracies for PP and CP obtained with the dominance model were both higher than those for PP obtained with the ASGM model.

#### Implications for Breeding Programs

All additive models for predicting CP rely on phenotypic data collected from crossbred animals. This can be problematic in situations where the crossbred animals are not individually identified and thus such data collection pipeline is not implemented. This is likely the case on many farms housing crossbred animals. While additive models require phenotypes from crossbred animals, this is not the case for dominance models because the breeding values for CP can be derived from additive and dominance effects that are predicted in the pure breed, and from the allele frequencies in the opposite breed. Esfandyari et al. (2015a) proposed therefore to use dominance models for selecting purebred animals for CP based on purebred phenotypic and genotypic information only. They did a simulation study and estimated the marker effects with Bayesian LASSO (Park and Casella, 2008; los Campos et al., 2009). The results showed that the gain in CP was higher when the purebreds were selected for CPs, which demonstrated the feasibility of the method even when no crossbred data is available. Moreover, combining several related lines into a single reference population increased the prediction accuracy. However, as shown by Esfandyari et al. (2015b), training on crossbred animals leads to a higher selection response than training on purebred animals. A likely explanation is, that the level of heterozygosity was higher than in the purebred data.

Although genomic selection for CP is a promising strategy to increase selection response for CP in the short and medium term, Esfandyari et al. (2018) found that genomic selection for CP leads eventually to lower CP in the long term than genomic selection on PP. This hold regardless of whether training was on purebred or crossbred animals.

### Dominance Model With Imprinting

Dominance effects, as well as additive effects may depend on the breed of origin, which may be due to imprinting or breed complementarity. It could therefore be advantageous to account for imprinting explicitly. A dominance model with imprinting needs to distinguish between the paternal and the maternal allele. If an animal has received allele *A*_1_ from line A and allele *A*_2_ from line B, then we denote its genotype as *A*_1_*A*_2_. The centered indicator matrix for genotype *A*_1_*A*_2_ is given by

$$\begin{array}{l} W_{X}^{A_{1}A_{2}}=H_{X}^{A_{1}A_{2}}-{\bar{H}}_{X}^{A_{1}A_{2}}, \end{array}$$

where $H_{Xim}^{A_{1}A_{2}}\in \left\{0,1\right\}$ equals one, if animal *i* from cross X has genotype *A*_1_*A*_2_ at SNP *m*, and ${\bar{H}}_{Xim}^{A_{1}A_{2}}$ is the proportion of animals from cross X that have this genotype at SNP *m*.

The dominance model with imprinting assumes that the vector $g_{X}$ with genotypic values of the crossbred animals has the representation

(8) $$\begin{array}{l} g_{X}=\mu_{X}1+\left(W_{X}^{21}+W_{X}^{22}\right)a_{A}^{X}+\left(W_{X}^{12}+W_{X}^{22}\right)a_{B}^{X}\\ \;+W_{X}^{21}d_{A}^{X}+W_{X}^{12}d_{B}^{X}, \end{array}$$

where $\mu_{X}$ is the population mean, vectors $a_{A}^{X}$ and $a_{B}^{X}$ contain breed-of-origin dependent additive effects, and vectors $d_{A}^{X}$ and $d_{B}^{X}$ contain breed-of-origin dependent dominance effects. The model equation for the crossbred animals becomes

(9) $$\begin{array}{l} y_{X}=X_{X}b_{X}+Z_{X}u_{X}+\left(W_{X}^{21}+W_{X}^{22}\right)a_{A}^{X}\\ \;+\left(W_{X}^{12}+W_{X}^{22}\right)a_{B}^{X}+W_{X}^{21}d_{A}^{X}+W_{X}^{12}d_{B}^{X}+E_{X}. \end{array}$$

If imprinting in the parental lines is neglected, then the model equations for the parental lines remain as in Equation (7). The vector with allele substitution effects for CP of line A is in this case

(10) $$\begin{array}{r} \begin{array}{c} \alpha^{A}=a_{A}^{X}+\left(1-p^{B}\right)\circ d_{A}^{X}-p^{B}\circ d_{B}^{X}, \end{array} \end{array}$$

where $p_{\;}^{B}$ is the vector with allele frequencies in the opposite line. The proof is given in the Supplementary Material. When the SNP effects in the cross do not depend on the breed of origin, then the model simplifies, and becomes identical to the dominance model with line-dependent effects.

Nishio and Satoh (2015) proposed two alternative parameterizations for models with dominance and imprinting and fitted them by assuming normally distributed SNP effects. Their first model includes an additive effect, a dominance effect, and an imprinting effect for the heterozygous genotype, while their second model includes a paternal and a maternal gametic effect, and a dominance effect. The models provided in a simulation study more accurate estimates of genotypic values than GBLUP. While the models of Nishio and Satoh (2015) have the advantage that only 3 effects are needed in the equivalent SNP model for modeling the contribution of each SNP to the genotypic value of an animal, the model in Equation (9) has the advantage that more rigorous prior assumptions can be made for the joint distribution of the effects. That is, if the paternal lines are closely related, then the additive effects $a_{A}^{X}$ and $a_{B}^{X}$ could assumed to be *a priori* highly correlated, as well as the dominance effects $d_{A}^{X}$ and $d_{B}^{X}$. However, the parameterization does not allow to predict the vectors $a_{A}^{X},a_{B}^{X},d_{A}^{X}$ and $d_{B}^{X}$ individually.

Esfandyari et al. (2015b) compared in a simulation study a Bayesian dominance model with imprinting with the corresponding dominance model with line-independent effects, but used a different parameterization. The model considered imprinting because it included a separate effect for each phased genotype. Compared to the model proposed above, it has the disadvantage that the effects have no direct interpretation as additive and dominance effects. The genetic effects of the parental breeds were a priori independent. Even though the authors did not simulate imprinting, they found that the dominance model with imprinting was superior, if the reference population was sufficiently large, and if both lines were not closely related. The reason may be that the LD between markers and QTL was different in the cross and in the parental lines, so the additive effects and dominance effects were population-dependent.

## Discussion

In this paper, genomic models for the analysis of discontinuous crossbred data were reviewed. Compared to purebred genomic models, the reviewed models were of much higher complexity due to the inclusion of dominance effects, breed-specific effects, imprinting effects, and the use of PP and/or CP data. In the following some additional aspects regarding the distribution of the SNP effects and the model choice are considered.

### Distribution of SNP Effects

The normal distribution is the most common assumption about the distribution of SNP effects. Such models have the advantage, that they have an equivalent representation as animal models with genomic covariance matrices for which fast solvers exist, such as DMU (Madsen et al., 2010), WOMBAT (Meyer, 2007), ASReml (Gilmour et al., 2009), blupf90 (Misztal, 1999), or MiX99 (Vuori et al., 2006). Although the assumption of a normal distribution is not likely to be fulfilled when large marker panels are used, the experience with purebred data suggest that the reliabilities of the breeding values are only slightly worse than those obtained with non-normally distributed marker effects. However, the situation in crossbreeding is different because the parental lines are commonly distantly related, and it may be envisaged to evaluate all lines simultaneously in order to increase the reliabilities of the breeding values. This requires that all QTL are in high LD with at least one marker, which implies the necessity to use a large marker panel. However, if the marker panel is large, then only few markers are needed to capture the effect of any QTL. Consequently, the true effects of most markers are actually zero. The model for genomic selection should account for this and assume as a prior distribution for the SNP effects a mixture of two distributions. One component provides the distribution for markers that are in strong LD with a QTL, and the other one is actually zero. In this case, a random-variable γ_*m*_ is commonly introduced, which indicates whether the effects of an SNP *m* are different from zero. Well-known examples are BayesB (Meuwissen et al., 2001), BayesC (Habier et al., 2011), and BayesR (Erbe et al., 2012). Such algorithms are usually implemented with MCMC algorithms, which results in long computation times. However, alternative and faster implementations are available for some models (e.g., Meuwissen, 2009; Shepherd et al., 2010).

For models with additive and dominance effects, an important aspect is, whether these effects are a priori independent or not. It may be advantageous to assume that all effects of a particular SNP *m* are of the same order of magnitude. This is possible if all effects of a particular SNP *m* have conditionally on the common covariance matrix $\gamma_{m}\sigma_{m}^{2}\Sigma$ a normal distribution, where $\sigma_{m}^{2}\;\sim \text{ Inv}$-χ^2^(*v, s*) and Σ is an appropriately chosen covariance matrix. For the dominance model with line-dependent effects, this means that

$$\begin{array}{l} \left(a_{m}^{A},a_{m}^{B},a_{m}^{X},d_{m}^{A},d_{m}^{B},d_{m}^{X}\right)|\sigma_{m}^{2},\gamma_{m}\sim N\left(0,\gamma_{m}\sigma_{m}^{2}\Sigma \right). \end{array}$$

It can be shown that in this case, all effects of SNP *m* would have for γ_*m*_ = 1 a *t*-distribution with *v* degrees of freedom, and are for γ_*m*_ = 0 equal to zero. Moreover, the magnitude of the effect size would be similar for all effects of a given SNP *m*, which reduces the proportion of overdominant SNP. Developing a fast algorithm for such a model is an area for future research.

### Model Choice

The most suitable model for a breeding program depends on the achievable accuracies for the breeding values of the selection candidates, and on the available data. Among the additive models, the parental model provided the least accurate predictions for CP, which is because the Mendelian sampling terms are part of the residual and can therefore not be utilized for prediction. It has, however, the advantage that the crossbred animals do not need to be genotyped and may therefore be suitable for animals with low economic value.

The BSAM and ASGM models provided similar results in most cases. The BSAM model, however, needs the trace of the alleles from the purebred parent breed to the crossbred end product, which is a source of potential errors. This might even be more a problem when more complex crossbred structures are involved, e.g., three- or four-way crossbred data. Vandenplas et al. (2016) and Sevillano et al. (2016) developed a statistical pipeline for this purpose and applied it to a three-way crossbred pig data set.

The reviewed papers suggest that the dominance models provide more accurate genomic breeding values for CP than the additive models. Although Xiang et al. (2016b) showed that this gain in accuracy results in the case of normally distributed SNP effects almost solely from capturing inbreeding depression, this may be not the case when large marker panels and appropriate Bayesian models are used for evaluation. Dominance models have the additional advantage that breeding values for crossbred performance can be obtained from purebred animals, so phenotyping and genotyping crossbred individuals may not be necessary. However, as shown by Esfandyari et al. (2015b), the accuracy of the breeding values can be increased when phenotyped and genotyped crossbred individuals are included in the reference population.

Three different dominance models have been applied to crossbred data, which are the dominance model with line-independent effects, the dominance model with line-dependent effects, and the dominance model with imprinting. The dominance model with line-dependent effects is likely to be inferior to the model with line-independent effects if the SNP effects of the different lines are falsely assumed to be statistically independent, the reference population is small, and the lines are closely related. This could be avoided by specifying a covariance between the SNP effects of the different lines.

When imprinting is relevant, then a dominance model with imprinting is of interest. For example, Jiang et al. (2017) found that there is substantial imprinting for reproduction traits in dairy cattle. The application of imprinting models requires that the crossbred animals are genotyped and that the alleles are traced from the parental lines to the crossbred animals. Unfortunately, to the best of our knowledge, these models are not well-analyzed yet. More research should be done in this area, which includes to analyze all models with common data sets.

## Conclusion

Genomic models for crossbred data are of much higher complexity than models for purebred data, which results from the inclusion of dominance effects, breed-specific effects, imprinting effects, and from the joint evaluation of PP and CP. Although much research has already been done to develop genomic models for crossbred data, it can be expected that further progress can be made by developing statistical models that include all the different genetic effects in a single model, assume realistic covariance structures between the genetic effects of different breeding lines, use large marker panels, and assume realistic distributions for the SNP effects. The comparisons made in the reviewed papers are not sufficiently comprehensive to come to a clear recommendation as to which existing method is most suitable for a specific breeding program and a specific genetic trait architecture. Some papers suggested a superiority of dominance models. In the reviewed papers, the focus was on discontinuous crossbreeding schemes. This was because, to our best knowledge, no genomic models have been published that are specifically designed for continuous crossbreeding schemes.

## Author Contributions

RW did the formal model comparison. JS, RW, and JB wrote the paper. DH contributed to the writing. All authors contributed to the article and approved the submitted version.

## Conflict of Interest

The authors declare that the research was conducted in the absence of any commercial or financial relationships that could be construed as a potential conflict of interest.
