# Supplementary material for: A Review of Genomic Models for the Analysis of Livestock Crossbred Data
Source: Front Genet. 2020 Jun 26;11:568. doi: 10.3389/fgene.2020.00568 (PMC7332767; doi:10.3389/fgene.2020.00568)
Supplement: Supplementary file 1 [file Data_Sheet_1.PDF]

The dominance model with imprinting

Robin Wellmann

February 2, 2020

The set of SNP is denoted as $\mathcal{M}$. All SNP are assumed to be biallelic and coded as alleles 1 and 2. A two-way cross $\mathcal{X}$ with sire line $\mathcal{A}$ and dam line $\mathcal{B}$ is considered. The cross is obtained by random mating of males from line $\mathcal{A}$ with females from line $\mathcal{B}$.

Definition 1. If a crossbred individual i has received allele A_1 from sire line $\mathcal{A}$ and allele A_2 from dam line $\mathcal{B}$, then its genotype is denoted as A_1A_2. The **centered genotype indicator** for genotype A_1A_2 is

$$W_{\mathcal{X}im}^{A_1A_2} = H_{\mathcal{X}im}^{A_1A_2} - \overline{H}_{\mathcal{X}m}^{A_1A_2},$$

where $H_{\mathcal{X}im}^{A_1A_2} \in \{0,1\}$ equals one, if individual i from cross $\mathcal{X}$ has genotype A_1A_2 at SNP m, and $\overline{H}_{\mathcal{X}m}^{A_1A_2}$ is the proportion of individuals from cross $\mathcal{X}$ with genotype A_1A_2 at SNP m.

Definition 2. The **dominance model with imprinting** assumes that the genotypic value of an individual i from cross $\mathcal{X}$ has the representation

$$g(i) = \mu_{\mathcal{X}} + \sum_{m\in\mathcal{M}} \left((W_{\mathcal{X}im}^{21} + W_{\mathcal{X}im}^{22})a_{\mathcal{A}m}^{\mathcal{X}} + (W_{\mathcal{X}im}^{12} + W_{\mathcal{X}im}^{22})a_{\mathcal{B}m}^{\mathcal{X}} + W_{\mathcal{X}im}^{21}d_{\mathcal{A}m}^{\mathcal{X}} + W_{\mathcal{X}im}^{12}d_{\mathcal{B}m}^{\mathcal{X}}\right),$$

where

- $a_{\mathcal{A}m}^{\mathcal{X}}$ is the additive effect of allele 2 at SNP m in cross $\mathcal{X}$ if allele 2 was received from line $\mathcal{A}$,
- $d_{\mathcal{A}m}^{\mathcal{X}}$ is the dominance effect of allele 2 at SNP m in cross $\mathcal{X}$ if allele 2 was received from line $\mathcal{A}$.

The SNP effects $a_{\mathcal{B}m}^{\mathcal{X}}$ and $d_{\mathcal{B}m}^{\mathcal{X}}$ are defined accordingly.

Lemma 1. In the dominance model with imprinting is $\mu_{\mathcal{X}}$ the mean genotypic value of cross $\mathcal{X}$.

Proof: Let individual i_b be randomly chosen from cross $\mathcal{X}$. Conditionally on the SNP effects, we have

$$
\begin{aligned}
& E(g(i_b)) \\
= \; & \mu_{\mathcal{X}} + \sum_{m \in \mathcal{M}} \left(E(W^{21}_{\mathcal{X}im} + W^{22}_{\mathcal{X}im}) a^{\mathcal{X}}_{\mathcal{A}m} + E(W^{12}_{\mathcal{X}im} + W^{22}_{\mathcal{X}im}) a^{\mathcal{X}}_{\mathcal{B}m} + E(W^{21}_{\mathcal{X}im}) d^{\mathcal{X}}_{\mathcal{A}m} + E(W^{12}_{\mathcal{X}im}) d^{\mathcal{X}}_{\mathcal{B}m} \right) \\
= \; & \mu_{\mathcal{X}} + \sum_{m \in \mathcal{M}} \left(0 a^{\mathcal{X}}_{\mathcal{A}m} + 0 a^{\mathcal{X}}_{\mathcal{B}m} + 0 d^{\mathcal{X}}_{\mathcal{A}m} + 0 d^{\mathcal{X}}_{\mathcal{B}m} \right) \\
= \; & \mu_{\mathcal{X}}
\end{aligned}
$$

□

Definition 3. The **centered allele content** of indivdiual i from line $\mathcal{A}$ is

$$Z^{\mathcal{A}}_{Cim} \;=\; C^{\mathcal{A}}_{im} - 2p^{\mathcal{A}}_{m},$$

where $C^{\mathcal{A}}_{im} \in \{0,1,2\}$ is the number of copies of allele 2, individual i has at SNP m.

Definition 4. The **breeding value** of individual i from line $\mathcal{A}$ **for crossbred performance** with respect to mating partners from line $\mathcal{B}$ is

$$c_{\mathcal{A}}(i) \;=\; 2(E(g(o_i)) - \mu_{\mathcal{X}}),$$

where the mating partner is chosen at random from line $\mathcal{B}$, and o_i is a randomly chosen offspring of individual i. The expectation is taken conditionally on the SNP effects.

Theorem 1. The breeding value of individual i from line $\mathcal{A}$ for crossbred performance with respect to mating partners from $\mathcal{B}$ is

$$c_{\mathcal{A}}(i) \quad = \quad \sum_{m \in \mathcal{M}} Z^{\mathcal{A}}_{Cim} \alpha^{\mathcal{X}}_{\mathcal{A}m},$$

where the allele substitution effects for crossbred performance are

$$\alpha^{\mathcal{X}}_{\mathcal{A}m} \quad = \quad a^{\mathcal{X}}_{\mathcal{A}m} + (1 - p^{\mathcal{B}}_m) d^{\mathcal{X}}_{\mathcal{A}m} - p^{\mathcal{B}}_m d^{\mathcal{X}}_{\mathcal{B}m}$$

for $m \in \mathcal{M}$.

Proof: Let o_i be a randomly chosen offspring of individual i from line $\mathcal{A}$. The individual has a paternal haplotype $\tilde{\mathcal{H}}_i$ and a maternal haplotype $\tilde{\mathcal{P}}_{\mathcal{B}}$, where

- haplotype $\tilde{\mathcal{H}}_i$ originates from animal i, and
- haplotype $\tilde{\mathcal{P}}_{\mathcal{B}}$ originates from population $\mathcal{B}$.

Let $\mathcal{H}_i = \tilde{\mathcal{H}}_i - 1$, $\mathcal{P}_{\mathcal{B}} = \tilde{\mathcal{P}}_{\mathcal{B}} - 1$, and $m \in \mathcal{M}$. Since $\mathcal{H}_{im}, \mathcal{P}_{\mathcal{B}m} \in \{0, 1\}$, the uncentered genotype indicators for offspring o_i are

$$\begin{aligned}
H^{11}_{\mathcal{X}o_im} &= (1 - \mathcal{H}_{im})(1 - \mathcal{P}_{\mathcal{B}m}) \\
H^{12}_{\mathcal{X}o_im} &= (1 - \mathcal{H}_{im})\mathcal{P}_{\mathcal{B}m} \\
H^{21}_{\mathcal{X}o_im} &= \mathcal{H}_{im}(1 - \mathcal{P}_{\mathcal{B}m}) \\
H^{22}_{\mathcal{X}o_im} &= \mathcal{H}_{im}\mathcal{P}_{\mathcal{B}m}.
\end{aligned}$$

Since the cross is obtained by random mating of individuals from line $\mathcal{A}$ with individuals from line $\mathcal{B}$, the centered genotype indicators are

$$\begin{aligned}
W^{11}_{\mathcal{X}o_im} &= (1 - \mathcal{H}_{im})(1 - \mathcal{P}_{\mathcal{B}m}) - (1 - p^{\mathcal{A}}_m)(1 - p^{\mathcal{B}}_m) \\
W^{12}_{\mathcal{X}o_im} &= (1 - \mathcal{H}_{im})\mathcal{P}_{\mathcal{B}m} - (1 - p^{\mathcal{A}}_m)p^{\mathcal{B}}_m \\
W^{21}_{\mathcal{X}o_im} &= \mathcal{H}_{im}(1 - \mathcal{P}_{\mathcal{B}m}) - p^{\mathcal{A}}_m(1 - p^{\mathcal{B}}_m) \\
W^{22}_{\mathcal{X}o_im} &= \mathcal{H}_{im}\mathcal{P}_{\mathcal{B}m} - p^{\mathcal{A}}_m p^{\mathcal{B}}_m.
\end{aligned}$$

For brevity, we write $W^{A_1A_2}_{o_im} = W^{A_1A_2}_{\mathcal{X}o_im}$ in the following. Conditionally on the SNP effects we have

$$\begin{aligned}
& E(g(o_i)) \\
&= E\left(\mu_{\mathcal{X}} + \sum_{m \in \mathcal{M}} \left((W^{21}_{o_im} + W^{22}_{o_im})a^{\mathcal{X}}_{\mathcal{A}m} + (W^{12}_{o_im} + W^{22}_{o_im})a^{\mathcal{X}}_{\mathcal{B}m} + W^{21}_{o_im}d^{\mathcal{X}}_{\mathcal{A}m} + W^{12}_{o_im}d^{\mathcal{X}}_{\mathcal{B}m}\right)\right) \\
&= \mu_{\mathcal{X}} + \sum_{m \in \mathcal{M}} \left(E(W^{21}_{o_im} + W^{22}_{o_im})a^{\mathcal{X}}_{\mathcal{A}m} + E(W^{12}_{o_im} + W^{22}_{o_im})a^{\mathcal{X}}_{\mathcal{B}m} + E(W^{21}_{o_im})d^{\mathcal{X}}_{\mathcal{A}m} + E(W^{12}_{o_im})d^{\mathcal{X}}_{\mathcal{B}m}\right).
\end{aligned}$$

Animal o_i carries with probability 0.5 the paternal allele and with probability 0.5 the maternal allele of parent i, so

$$E(\mathcal{H}_{im}) = \frac{C^{\mathcal{A}}_{im}}{2},$$

and

$$E(\mathcal{H}_{im}) - p^{\mathcal{A}}_{m} = \frac{Z^{\mathcal{A}}_{Cim}}{2}.$$

Thus,

$$\begin{aligned}
E(W^{21}_{o_im} + W^{22}_{o_im}) &= E\left(\mathcal{H}_{im}(1-\mathcal{P}_{\mathcal{B}m}) - p^{\mathcal{A}}_{m}(1-p^{\mathcal{B}}_{m}) + \mathcal{H}_{im}\mathcal{P}_{\mathcal{B}m} - p^{\mathcal{A}}_{m}p^{\mathcal{B}}_{m}\right)\\
&= E\left(\mathcal{H}_{im} - p^{\mathcal{A}}_{m}\right)\\
&= \frac{Z^{\mathcal{A}}_{Cim}}{2},\\
E(W^{12}_{o_im} + W^{22}_{o_im}) &= E\left((1-\mathcal{H}_{im})\mathcal{P}_{\mathcal{B}m} - (1-p^{\mathcal{A}}_{m})p^{\mathcal{B}}_{m} + \mathcal{H}_{im}\mathcal{P}_{\mathcal{B}m} - p^{\mathcal{A}}_{m}p^{\mathcal{B}}_{m}\right)\\
&= E\left(\mathcal{P}_{\mathcal{B}m} - p^{\mathcal{B}}_{m}\right)\\
&= 0,
\end{aligned}$$

Since the mating partner of indivdiual i was chosen at random from line $\mathcal{B}$, we have

$$\begin{aligned}
E(W^{21}_{o_im}) &= E\left(\mathcal{H}_{im}(1-\mathcal{P}_{\mathcal{B}m}) - p^{\mathcal{A}}_{m}(1-p^{\mathcal{B}}_{m})\right)\\
&= E(\mathcal{H}_{im})(1-p^{\mathcal{B}}_{m}) - p^{\mathcal{A}}_{m}(1-p^{\mathcal{B}}_{m})\\
&= E(\mathcal{H}_{im} - p^{\mathcal{A}}_{m})(1-p^{\mathcal{B}}_{m})\\
&= \frac{Z^{\mathcal{A}}_{Cim}}{2}(1-p^{\mathcal{B}}_{m}),\\
E(W^{12}_{o_im}) &= E\left((1-\mathcal{H}_{im})\mathcal{P}_{\mathcal{B}m} - (1-p^{\mathcal{A}}_{m})p^{\mathcal{B}}_{m}\right)\\
&= (1-E(\mathcal{H}_{im}))p^{\mathcal{B}}_{m} - (1-p^{\mathcal{A}}_{m})p^{\mathcal{B}}_{m}\\
&= (p^{\mathcal{A}}_{m} - E(\mathcal{H}_{im}))p^{\mathcal{B}}_{m}\\
&= (E(\mathcal{H}_{im}) - p^{\mathcal{A}}_{m})(-p^{\mathcal{B}}_{m})\\
&= \frac{Z^{\mathcal{A}}_{Cim}}{2}(-p^{\mathcal{B}}_{m}).
\end{aligned}$$

Thus,

$$\begin{aligned}
E(g(o_i)) &= \mu_{\mathcal{X}} + \sum_{m\in\mathcal{M}}\left(\frac{Z^{\mathcal{A}}_{Cim}}{2}a^{\mathcal{X}}_{\mathcal{A}m} + 0a^{\mathcal{X}}_{\mathcal{B}m} + \frac{Z^{\mathcal{A}}_{Cim}}{2}(1-p^{\mathcal{B}}_{m})d^{\mathcal{X}}_{\mathcal{A}m} + \frac{Z^{\mathcal{A}}_{Cim}}{2}(-p^{\mathcal{B}}_{m})d^{\mathcal{X}}_{\mathcal{B}m}\right)\\
&= \mu_{\mathcal{X}} + \sum_{m\in\mathcal{M}}\frac{Z^{\mathcal{A}}_{Cim}}{2}\left(a^{\mathcal{X}}_{\mathcal{A}m} + (1-p^{\mathcal{B}}_{m})d^{\mathcal{X}}_{\mathcal{A}m} + (-p^{\mathcal{B}}_{m})d^{\mathcal{X}}_{\mathcal{B}m}\right)\\
&= \mu_{\mathcal{X}} + \frac{1}{2}\sum_{m\in\mathcal{M}} Z^{\mathcal{A}}_{Cim}\left(a^{\mathcal{X}}_{\mathcal{A}m} + (1-p^{\mathcal{B}}_{m})d^{\mathcal{X}}_{\mathcal{A}m} - p^{\mathcal{B}}_{m}d^{\mathcal{X}}_{\mathcal{B}m}\right).
\end{aligned}$$

□
